# Mechanical performance of original; yellowish and blueish ProFile instruments: isolating heat-treatment as a variable

**DOI:** 10.1590/0103-6440202204978

**Published:** 2022-08-26

**Authors:** Edson J. L. Moreira, Emmanuel J.N.L. Silva, Felipe G. Belladonna, Ana Carolina Maciel, Victor T. L. Vieira, Gustavo De-Deus

**Affiliations:** 1 Department of Endodontics; Fluminense Federal University (UFF); Niterói; RJ; Brazil

**Keywords:** Endodontic, instruments, NiTi, mechanical properties

## Abstract

The present study aimed to perform two different heat-treatments in an austenitic NiTi ProFile instrument and to compare the mechanical performance of original and heat-treated instruments. Heat treatment of ProFile (tip size 25 and 0.06 taper) instruments were carried out in a furnace in argon atmosphere using a heating rate of 10° C/min. After reaching the programmed temperatures of 450 ºC or 500 ºC the system remained at a constant temperature for 10 minutes; followed by cooling in water at room temperature. Afterwards; the three groups (n=30 per group) of instruments were compared regarding their cyclic fatigue (n=10 per group); bending (n=10 per group); and buckling resistance (n=10 per group). After cyclic fatigue tests; a scanning electron microscope was used to analyze the fracture surfaces and observe the fracture mode. Statistical analysis was performed using One-way ANOVA and Student-Newman-Keuls test; with an alpha type error set at 0.05. Yellowish and blueish coloration was observed in the ProFile instruments after 450 ºC or 500 ºC heat treatments; respectively. Conventional ProFile instruments showed the lower cyclic fatigue; and the higher bending and buckling resistance (P<0.05). In contrast; yellowish ProFile instruments (heat treated at 500° C) showed the higher cyclic fatigue; and the lower bending and buckling resistance (P>0.05). It can be concluded that the different heat treatments performed on ProFile instruments increased its cyclic fatigue resistance and improved the flexibility and buckling resistance.

## Resumo

O presente estudo teve como objetivo realizar dois tratamentos térmicos diferentes em instrumentos de NiTi austenítico ProFile e comparar o desempenho mecânico de instrumentos originais e tratados termicamente. O tratamento térmico dos instrumentos ProFile (tamanho de ponta 25 e conicidade 0.06) foi realizado em um forno em atmosfera de argônio usando uma taxa de aquecimento de 10°C/min. Após atingir as temperaturas programadas de 450 ºC ou 500 ºC o sistema permaneceu em temperatura constante por 10 minutos; seguido de resfriamento em água à temperatura ambiente. Em seguida; os três grupos de instrumentos (n=30 por grupo) foram comparados quanto à fadiga cíclica (n=10 por grupo); flexão (n=10 por grupo) e resistência à flambagem (n=10 por grupo). Após testes de fadiga cíclica; um microscópio eletrônico de varredura foi utilizado para analisar as superfícies de fratura e observar o modo de fratura. A análise estatística foi realizada por meio dos testes de One-way ANOVA e teste de Student-Newman-Keuls; com erro de tipo alfa fixado em 0.05. Coloração amarelada e azulada foi observada nos instrumentos ProFile após tratamentos térmicos a 450 ºC ou 500 ºC; respectivamente. Os instrumentos ProFile originais apresentaram menor fadiga cíclica e maior resistência à flexão e flambagem (P<0.05). Em contraste; os instrumentos ProFile azulados (tratados termicamente a 500°C) apresentaram maior fadiga cíclica e menor resistência à flexão e flambagem (P>0.05). Pode-se concluir que os diferentes tratamentos térmicos realizados nos instrumentos ProFile aumentaram sua resistência à fadiga cíclica e melhoraram a flexibilidade e resistência à flambagem.

## Introduction

The introduction of NiTi endodontic instruments in clinical practice has resulted in an overall improvement in the treatment quality, allowing for safer mechanized instrumentation while maintaining the original conformation of the root canals canal ^1^^,^^2^^,^^3^. The intrinsic characteristics of the NiTi alloy enabled the development of instruments prone to better control the occurrence of ordinary iatrogenic mishaps such as deviation and perforation ^4^. In fact, it is fair to say that shaping outcomes with NiTi rotary instruments are more predictable, and that canal enlargement procedure is simpler and more pleasant for dentists when compared to the manual preparation with stainless-steel files ^5^^,^^6^. 

Despite the numerous advantages of NiTi instruments, these instruments still presents risk of fracture, especially during its use in curved canals, which might compromise the prognosis of root canal treatment ^7^. To overcome the fracture problems, innovations have been proposed to improve instruments flexibility and fracture resistance, such as changes in the geometry of the cross section, changes in the applied kinematics, improvements in the surface finish, and the use of thermal treatment of NiTi alloys ^8^^,^^9^^,^^10^. The performance of thermal treatments promotes significant changes in the NiTi alloy's intrinsic characteristics and mechanical properties presented by the instruments, highlighting local changes in the chemical composition, structural phase transformation, change in phase transition temperatures, in addition to promoting a significant increase in flexibility and resistance to fatigue presented by endodontic instruments ^6^^,^^11^^,^^12^^,^^13^. 

Despite a vast report in the literature on the improvements resulting from heat treatments in endodontic NiTi instruments ^6^^,^^8^^,^^12^^,^^14^, no study evaluated the direct influence of temperature and treatment time on instruments properties. Moreover, the design of most studies does not allow isolating only heat treatment as a variable, since instruments with different tips, tapers, cross-sectional design and manufacturing and finishing methods are compared. It is well known that these other factors can directly influence in the results of the mechanical properties of an instrument. Therefore, the present study aimed to perform different heat-treatments in a conventional austenitic ProFile (Dentsply Maillefer, Baillagues, Switzerland) NiTi endodontic instrument and to compare the cyclic fatigue, bending and buckling resistance of original instrument with those obtained after heat-treatments. The null hypothesis tested was that the thermal treatment does not interfere in the mechanical properties of the NiTi ProFile instruments. 

## Materials and methods

### Sample size calculation

The sample size was estimated based on the results of the different instruments after the 5 initial measurements. The ANOVA, fixed effects, omnibus, One-way test, was selected from the F-tests family in G*Power 3.1 software for Windows (Henrick Heine-Universitat, Dusseldorf, Germany). For the cyclic fatigue analysis with a = 0.05 and 95% testing power, and considering an effect size = 41, a total of two specimens were indicated as the ideal size required for observing significant differences. For the bending resistance analysis with a = 0.05 and 95% testing power, and considering an effect size = 49, a total of two specimens were indicated as the ideal size required for observing significant differences. Finally, for the buckling resistance analysis with a = 0.05 and 95% testing power, and considering an effect size = 45, a total of two specimens were indicated as the ideal size required for observing significant differences. Ten instruments were allocated in each testing group and for each performed test. 

### Instruments used

Ninety new 25-mm NiTi ProFile (tip size 25 and 0.06 taper) instruments were used. Before any assessment, the instruments were visually inspected under a x13.6 magnification (Opmi Pico, Carl Zeiss Surgical, Germany) looking for defects that would exclude them from being tested; however, none was excluded. 

### Heat treatments and group division

**Figure 1:**
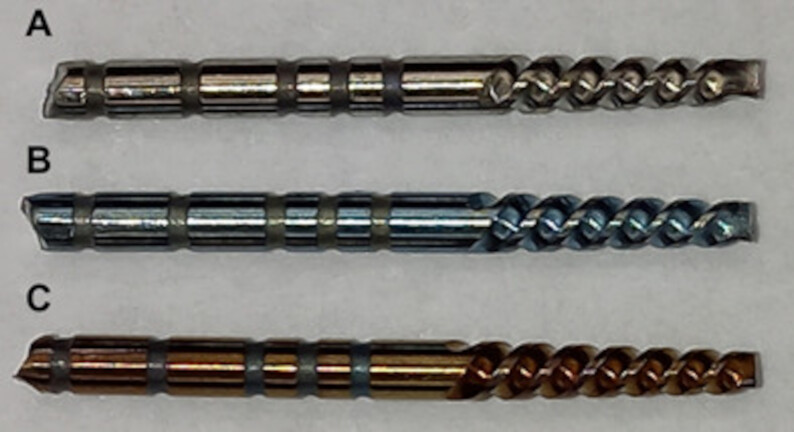
Images of (A) Conventional ProFile instruments, (B) Blueish ProFile instruments after 500 ºC heat treatments, and (C) Yellowish ProFile instruments after 450 ºC heat treatments.

Heat treatments of ProFile instruments were carried out in the NBD-O1200 furnace (Nobody Material Science and Technology CO, Xin Cun, Henan, China). The heat treatment was carried out with a heating rate of 10 °C/min. After reaching the programmed temperatures of 450 ºC and 500 ºC the system remained at a constant temperature for 10 minutes, followed by cooling in water at room temperature. The heat treatment was carried out in an argon atmosphere. The 450 ºC provided the formation of a golden oxide layer on the surface of the instrument, while the 500 ºC treatment provided a blue coloration (Figure 1). 

### Cyclic fatigue test

A non-tapered custom-made stainless-steel tube model apparatus was used for the cyclic fatigue test as reported in previous studies ^10^^,^^12^^,^^14^. Ten instruments of each type (original Profile, yellowish Profile - heat-treat at 450° C, and blueish Profile - heat-treated at 500° C) were activated in a continuous clockwise rotation by a 6:1 reduction handpiece (Sirona Dental Systems GmbH, Bensheim, Germany) powered by a torque-controlled motor (VDW Silver; VDW GmbH, Munich, Germany) at a static position using glycerin as lubricant. The time to fracture (in seconds) was established when the fracture was detected by visual and auditory inspection, and the size (in mm) of the fractured segments recorded using a digital caliper (Mitutoyo) for experimental control. 

After tests, a scanning electron microscope (SEM) (JSM 5800; JEOL, Tokyo, Japan) was used to analyze the fracture surfaces of all the tested instruments in order to observe the fracture mode. Different magnifications were used (× 250, and × 1500).

### Bending test

Ten instruments of each type (original Profile, yellowish Profile - heat-treat at 450° C, and blueish Profile - heat-treated at 500° C) were used for the bending test. The instruments mounted in a file holder and positioned at 45º to the floor, while their apical 3 mm were attached to a wire connected to an Universal Testing Machine (Instron EMIC DL-200 MF, São José dos Pinhais, Brazil), until a 45º displacement occurs under a 20 N load at 15 mm/min constant speed. The maximum load needed for the displacement was recorded in gram/force (gf).

### Buckling resistance test

Ten instruments of each type (original Profile, yellowish Profile - heat-treat at 450° C, and blueish Profile - heat-treated at 500° C) was tested for the buckling resistance test. For this, the load was applied in the axial direction of each instrument using a universal testing machine (Instron EMIC DL-200 MF). The maximum buckling load (elastic lateral deformation) was performed according to previously published studies ^15^^,^^16^. A 20 N loading cell was used. The mounting rod of the instrument was attached to the head of the universal testing machine by a chuck, and the tip of the instrument was compressed under an aluminum plate with a rough surface. The test was carried out at a speed of 1 mm min^-1^. The force was applied in the axial direction until a lateral elastic (compressive) displacement of 1 mm occurred, when the force was registered. 

### Statistical analysis

Because of the preliminary analysis of the raw pooled and isolated data revealed a bell-shaped distribution (Shapiro-Wilk normality test), statistical analysis was performed using parametric methods (one-way ANOVA). Post hoc pairwise comparisons were performed by using the Student-Newman-Keuls test. The alpha type error was set at 0.05. The Primer of Biostatistics program version 6.0 (McGraw-Hill, New York, NY, USA) ran all tests.

## Results

Yellowish and blueish coloration was observed in the ProFile instruments after 450 ºC or 500 ºC heat treatments, respectively (FIGURE 1). Conventional ProFile instruments showed the lower cyclic fatigue, and the higher bending and buckling resistance (P<0.05). In contrast, yellowish ProFile instruments showed the higher cyclic fatigue, and the lower bending and buckling resistance (P>0.05) (Table 1). 

**Table 1:** Mean and standard deviation of cyclic fatigue resistance (in sec), bending resistance (gf), and buckling resistance (gf).

Instrument	Cyclic fatigue (in sec)	Bending resistance (gf)	Buckling resistance (gf)
Original ProFile	12±3^A^	626±20^A^	502±37^A^
Blueish ProFile	76±8^B^	385±24^B^	268±12^B^
Yellowish ProFile	109±21^C^	269±17^C^	176±11^C^

Different superscript letters indicate significant differences amongst groups in the same column (P < 0.05).

**Figure 2:**
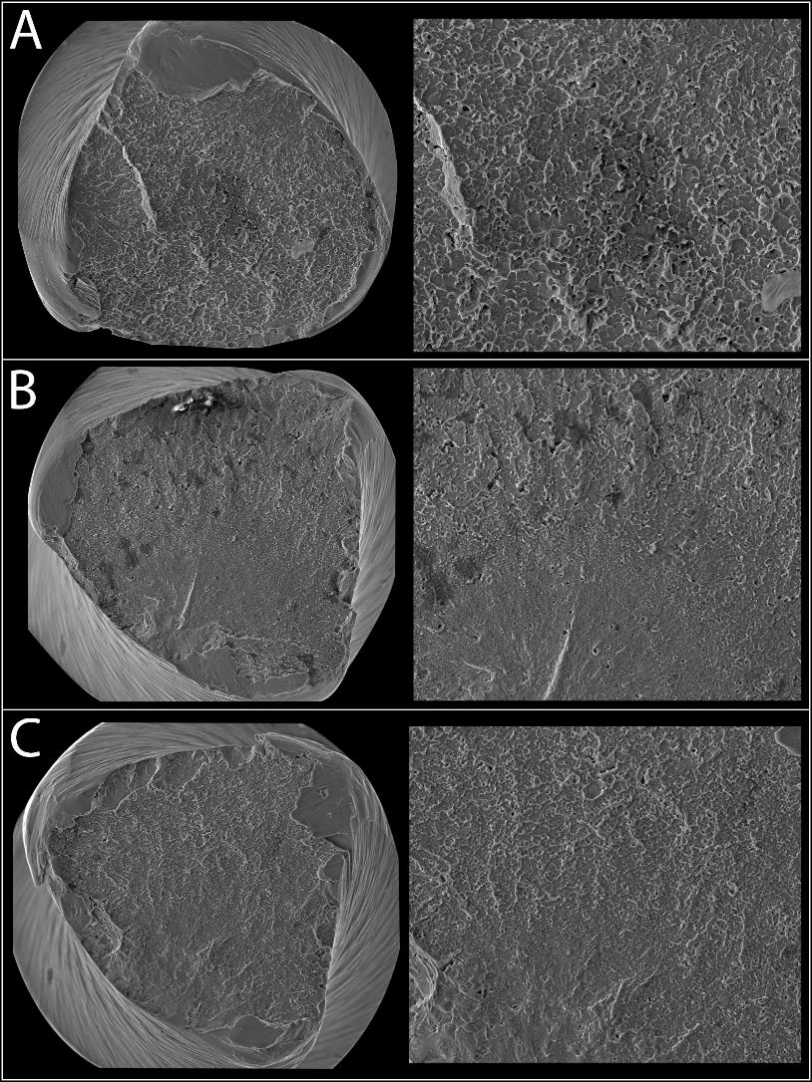
Representative SEM imagens of fractured surfaces of (A) Conventional ProFile, (B) Yellowish ProFile, and (C) Blueish ProFile instruments after cyclic fatigue test (x250 and x1500). The fractured surfaces showed morphologic characteristics of the ductile type with numerous dimples.

Scanning electron microscopy of the fracture surface showed similar and typical features of cyclic fatigue for the three different tested instruments. The fractured surfaces showed morphologic characteristics of the ductile type with numerous dimples. The crack initiation area and overload fast fracture zone for cyclic fatigue can be observed in the Figure 2. 

## Discussion

The present study compared the mechanical performance of conventional and heat-treated Profile instruments, through the evaluation of cyclic fatigue, bending and buckling resistance. Several studies comparing the mechanical properties of commercially available NiTi instruments with different heat-treatments are found in the literature ^3^^,^^9^^,^^10^^,^^12^^,^^14^. However, most of these studies compared instruments with different geometries (cross section, tip and taper). Despite these studies are relevant as they compare widely used instruments, it is well known that such variables have a direct influence on the mechanical properties of NiTi instruments. In the present study, influence of instrument design on the mechanical properties was virtually eliminated as the same instrument was tested, differing only in their thermal treatment. For this, ProFile NiTi endodontic instrument which is manufactured with conventional austenitic NiTi alloy and have been prevailing in the market since the year 1996 was selected. Similar set-up, eliminating the design influence on the mechanical properties, was previously used in studies that compared conventional austenitic alloys with “gold alloy” ^17^ and also M-wire with “blue-alloy” ^8^. However, to the author’s knowledge, there are no studies to date comparing conventional, gold and blue alloys. The overall results of the present study showed striking differences for all evaluated parameters and for all tested groups; therefore, the tested null hypothesis was rejected. 

To carry out this comparison, this study performed heat treatments on Profile tip 25 and 0.06 taper instruments, until yellowish and blueish colorations were obtained on the surface of the instruments. This coloration occurs due to the formation of an oxide layer that varies in thickness according to the time and temperature of the treatment ^13^. The thickness of the oxide layer acts as a filter for white light and generates a characteristic coloration in the instruments. More important than the oxide layer is the internal microstructure of the material that traps martensite ^13^, a phase with a lower modulus of elasticity, which justifies the striking differences observed in the present study.

Quantifying martensite is not feasible in irregular specimens such as endodontic instruments. The most suitable technique for this would be X-ray diffraction analysis, using Hetfield analysis, but for that a flat surface would be needed, requiring a preparation that could modify the surface microstructure. The coloration of the oxide layer on the instruments surface is evidence of the amount of martensite trapped in the microstructure and the cyclic fatigue life, bending and buckling resistance observed in the present study contributes to prove the differences in the final amount of martensite. 

In the present study, the instruments were tested for bending and buckling resistance using international guidelines and based on previously published studies ^12^^,^^14^^,^^16^^,^^18^. As cyclic fatigue resistance test does not have a specification or an international standard to evaluate NiTi endodontic instruments, this test was performed in the present study using a methodology validated in different studies published in peer-reviewed journals ^8^^,^^9^^,^^10^^,^^12^^,^^14^^,^^15^. Considering that instruments have the same design, it is not expected that the cyclic fatigue setup used in the present study can have a major influence in the current results. 

The results from the bending and buckling resistance test of the present study showed that heat-treated instruments had a significant improvement in flexibility and buckling resistance over non-treated Profile instruments. These results confirm those obtained in several previous studies that demonstrated lower flexibility and buckling resistance of austenitic NiTi alloy when compared to martensitic instruments ^17^. The non-treated instruments have supereslastic characteristic of a predominantly austenitic instrument ^19^. Due to the higher modulus of elasticity of austenite, these instruments show greater structural rigidity, which explains their lower flexibility and bending resistance compared to heat-treated ones. Austenitic and martensitic crystallographic arrangements may present distinct behaviors ^13^^,^^20^. It has been previously demonstrated that heat treatment of NiTi instruments improve their flexibility, making them more effective for preparation of curved canals ^21^. Therefore, clinically, heat treated instruments might have better shaping ability in curved root canals when compared to austenitic NiTi instruments. 

The results of the cyclic fatigue test are strictly related with the flexibility of the instruments tested and confirmed previous studies in which more flexible alloys showed enhanced cyclic fatigue ^8^^,^^14^^,^^15^^,^^22^^,^^23^. In fact, both heat-treated instruments showed a significant increase in the time to fracture when compared with the traditional Profile instruments. The finding of this study corroborates the literature that demonstrates that thermally treated instruments have a longer fatigue life when compared to austenitic instruments ^8^^,^^17^. The longer time of the thermally treated instruments can be explained by the lower tension levels generated inside the simulated canal. Following the implementation of heat treated NiTi instruments, the instruments became safer with a good performance in shaping ability, especially in preparation with anatomical challenges such as curved root canals, with clinical studies reporting low fracture rates ^24^^,^^25^. Such outcomes are in accordance with the current results that demonstrated longer cyclic fatigue life of these martensitic NiTi alloys when compared to austenitic alloys. 

Some experimental techniques commonly used in studies such as energy-dispersive X-ray spectroscopy and Vickers microhardness were not considered for this study for some reasons. The energy-dispersive X-ray spectroscopy was not used as the chemical composition of the instruments was not modified. As with X-ray diffraction analysis, Vickers microhardness presents critical sample preparation since the instruments would have to be embedded and sanded, and this would influence the fraction of martensite trapped in the heat treatment acting as a methodological bias.

Taken together, it can be concluded that the different heat treatments performed on ProFile instruments increased its cyclic fatigue resistance and improved the flexibility and buckling resistance. For this reason, the tested null hypothesis was rejected. 
